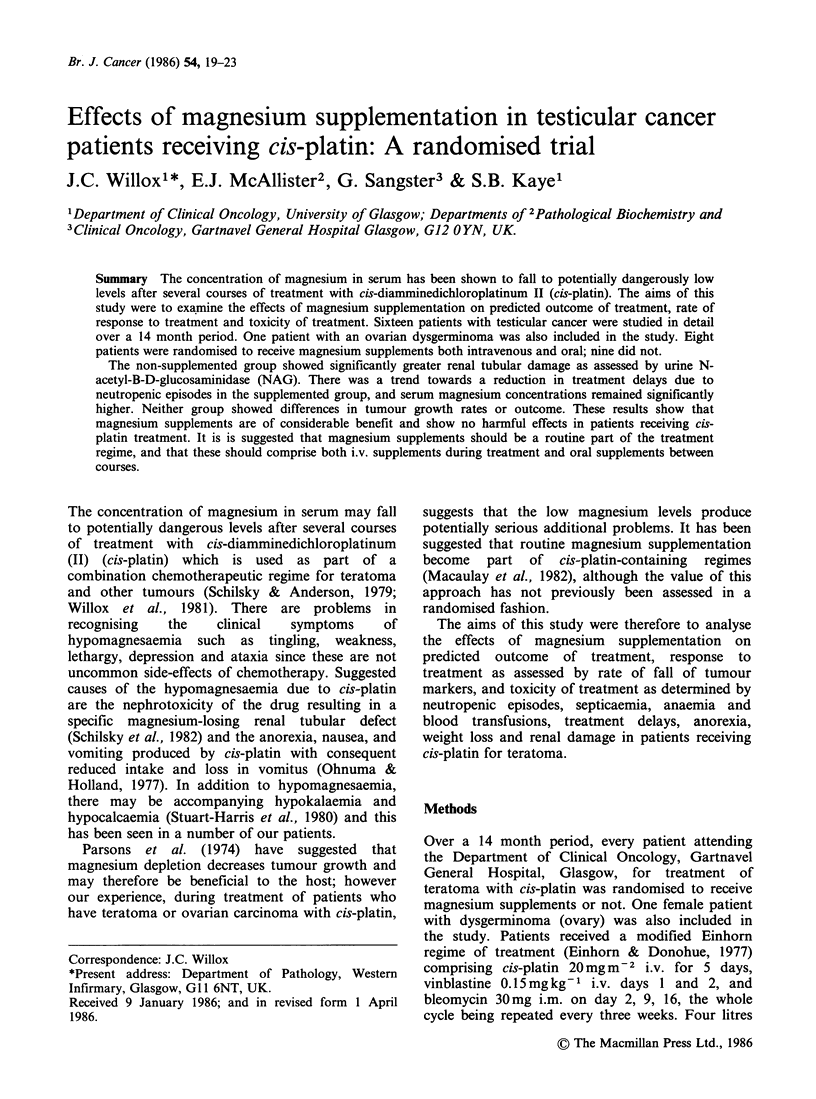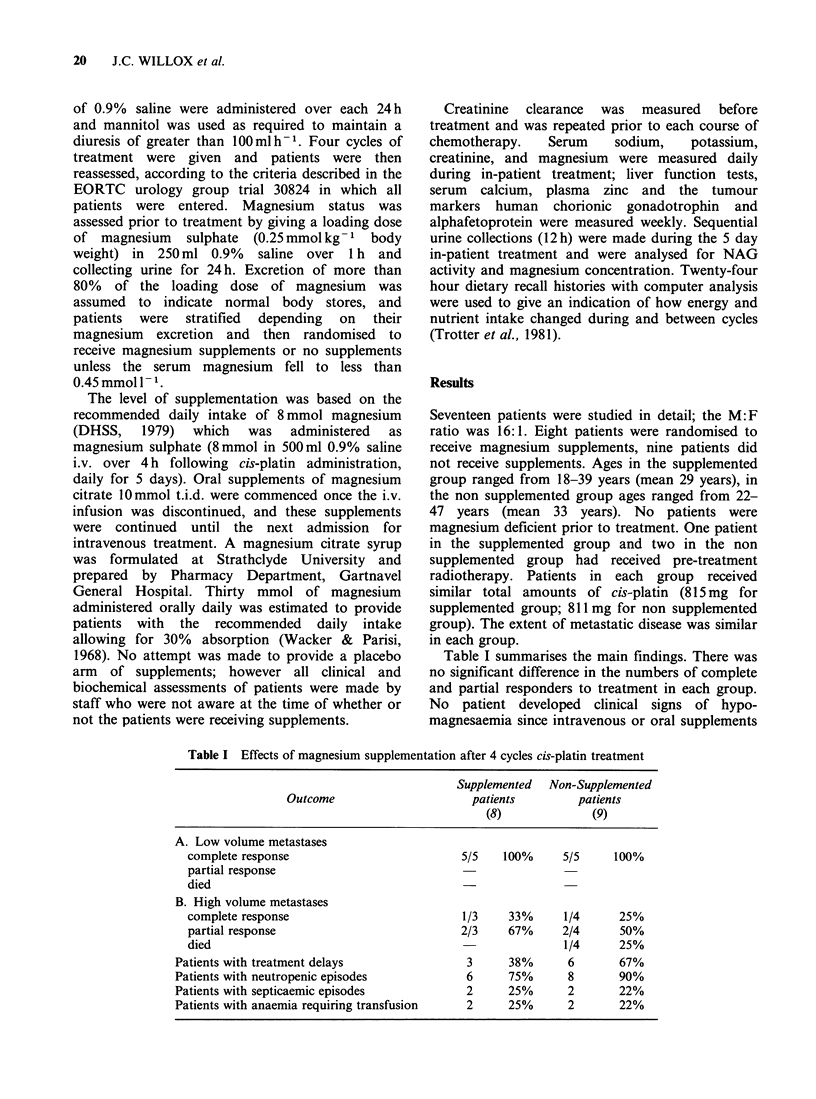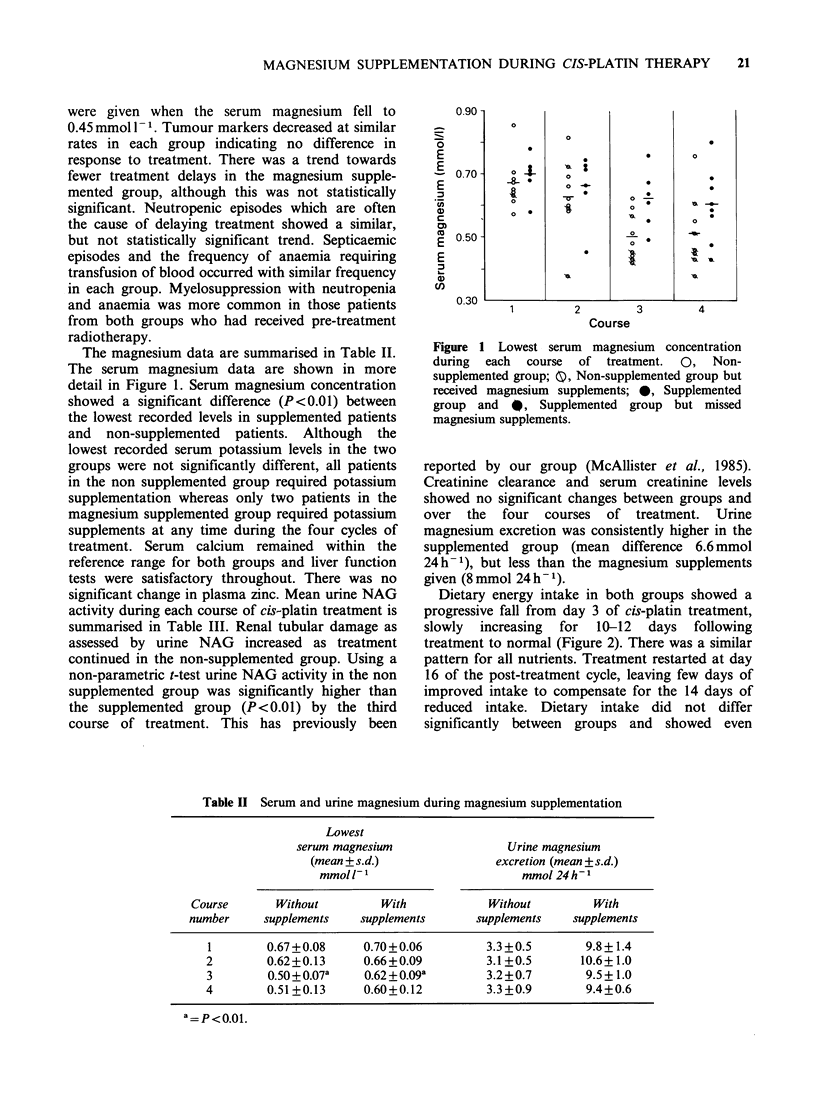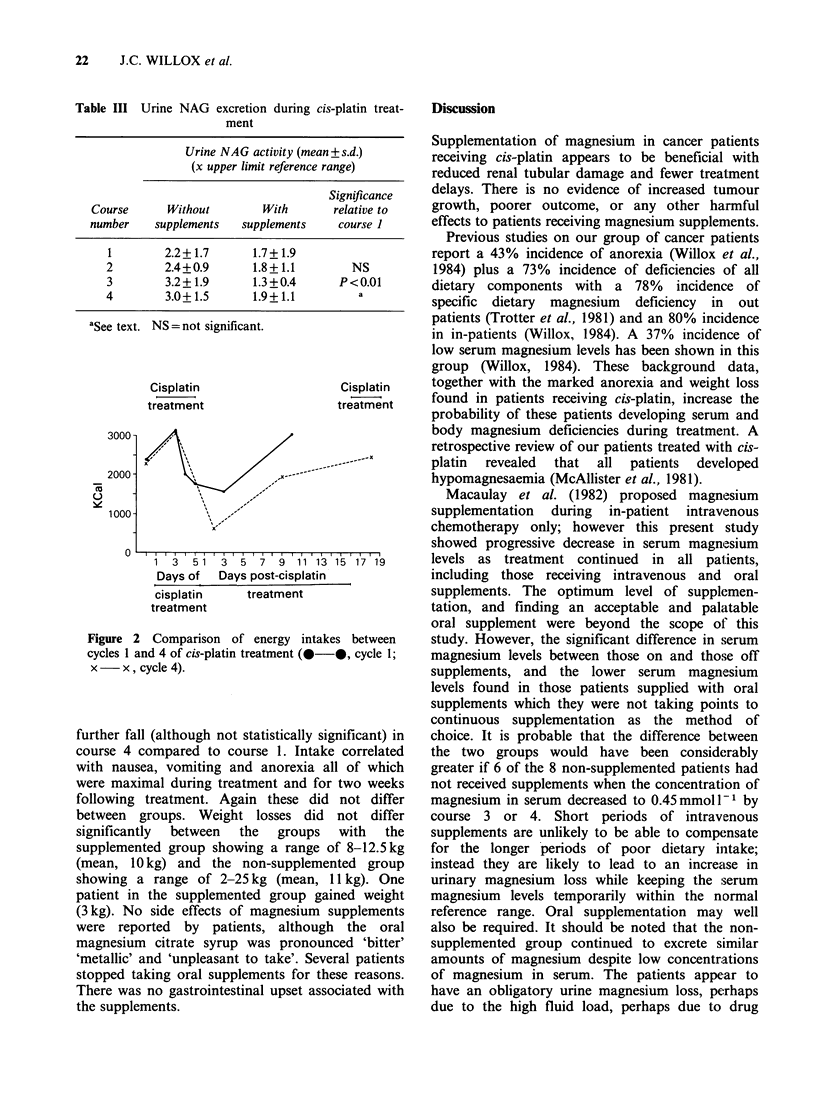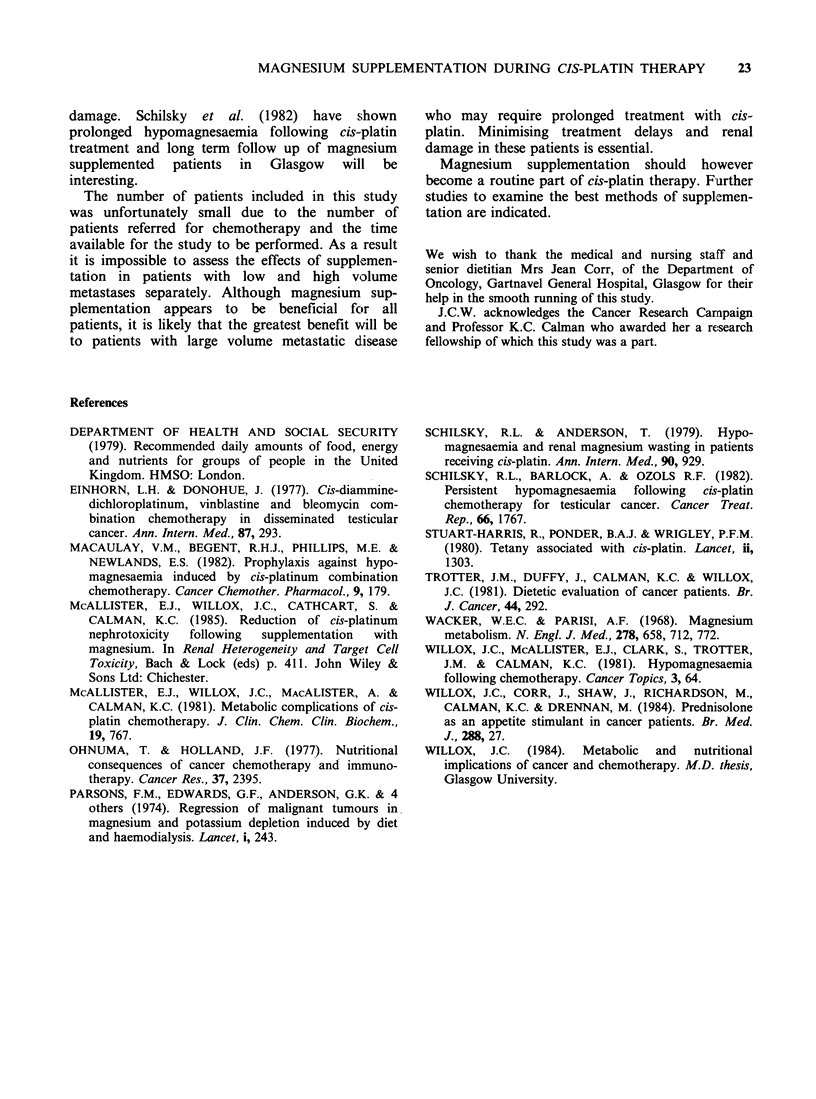# Effects of magnesium supplementation in testicular cancer patients receiving cis-platin: a randomised trial.

**DOI:** 10.1038/bjc.1986.147

**Published:** 1986-07

**Authors:** J. C. Willox, E. J. McAllister, G. Sangster, S. B. Kaye

## Abstract

The concentration of magnesium in serum has been shown to fall to potentially dangerously low levels after several courses of treatment with cis-diamminedichloroplatinum II (cis-platin). The aims of this study were to examine the effects of magnesium supplementation on predicted outcome of treatment, rate of response to treatment and toxicity of treatment. Sixteen patients with testicular cancer were studied in detail over a 14 month period. One patient with an ovarian dysgerminoma was also included in the study. Eight patients were randomised to receive magnesium supplements both intravenous and oral; nine did not. The non-supplemented group showed significantly greater renal tubular damage as assessed by urine N-acetyl-B-D-glucosaminidase (NAG). There was a trend towards a reduction in treatment delays due to neutropenic episodes in the supplemented group, and serum magnesium concentrations remained significantly higher. Neither group showed differences in tumour growth rates or outcome. These results show that magnesium supplements are of considerable benefit and show no harmful effects in patients receiving cis-platin treatment. It is is suggested that magnesium supplements should be a routine part of the treatment regime, and that these should comprise both i.v. supplements during treatment and oral supplements between courses.


Br. J. Cancer (1986) 54, 19–23

### Effects of magnesium supplementation in testicular cancer patients receiving *cis*-platin: A randomised trial

J.C. Willox[1]*, E.J. McAllister[2], G. Sangster[3] & S.B. Kaye[1]

[1]*Department of Clinical Oncology, University of Glasgow; Departments of* [2]*Pathological Biochemistry and* [3]*Clinical Oncology, Gartnavel General Hospital Glasgow, G12 0YN, UK.*

**Summary** The concentration of magnesium in serum has been shown to fall to potentially dangerously low levels after several courses of treatment with *cis*-diamminedichloroplatinum II (*cis*-platin). The aims of this study were to examine the effects of magnesium supplementation on predicted outcome of treatment, rate of response to treatment and toxicity of treatment. Sixteen patients with testicular cancer were studied in detail over a 14 month period. One patient with an ovarian dysgerminoma was also included in the study. Eight patients were randomised to receive magnesium supplements both intravenous and oral; nine did not.

The non-supplemented group showed significantly greater renal tubular damage as assessed by urine N-acetyl-B-D-glucosaminidase (NAG). There was a trend towards a reduction in treatment delays due to neutropenic episodes in the supplemented group, and serum magnesium concentrations remained significantly higher. Neither group showed differences in tumour growth rates or outcome. These results show that magnesium supplements are of considerable benefit and show no harmful effects in patients receiving *cis*-platin treatment. It is is suggested that magnesium supplements should be a routine part of the treatment regime, and that these should comprise both i.v. supplements during treatment and oral supplements between courses.

The concentration of magnesium in serum may fall to potentially dangerous levels after several courses of treatment with *cis*-diamminedichloroplatinum (II) (*cis*-platin) which is used as part of a combination chemotherapeutic regime for teratoma and other tumours (Schilsky & Anderson, 1979; Willox *et al.*, 1981). There are problems in recognising the clinical symptoms of hypomagnesaemia such as tingling, weakness, lethargy, depression and ataxia since these are not uncommon side-effects of chemotherapy. Suggested causes of the hypomagnesaemia due to *cis*-platin are the nephrotoxicity of the drug resulting in a specific magnesium-losing renal tubular defect (Schilsky *et al.*, 1982) and the anorexia, nausea, and vomiting produced by *cis*-platin with consequent reduced intake and loss in vomitus (Ohnuma & Holland, 1977). In addition to hypomagnesaemia, there may be accompanying hypokalaemia and hypocalcaemia (Stuart-Harris *et al.*, 1980) and this has been seen in a number of our patients.

Parsons *et al.* (1974) have suggested that magnesium depletion decreases tumour growth and may therefore be beneficial to the host; however our experience, during treatment of patients who have teratoma or ovarian carcinoma with *cis*-platin, suggests that the low magnesium levels produce potentially serious additional problems. It has been suggested that routine magnesium supplementation become part of *cis*-platin-containing regimes (Macaulay *et al.*, 1982), although the value of this approach has not previously been assessed in a randomised fashion.

The aims of this study were therefore to analyse the effects of magnesium supplementation on predicted outcome of treatment, response to treatment as assessed by rate of fall of tumour markers, and toxicity of treatment as determined by neutropenic episodes, septicaemia, anaemia and blood transfusions, treatment delays, anorexia, weight loss and renal damage in patients receiving *cis*-platin for teratoma.

### Methods

Over a 14 month period, every patient attending the Department of Clinical Oncology, Gartnavel General Hospital, Glasgow, for treatment of teratoma with *cis*-platin was randomised to receive magnesium supplements or not. One female patient with dysgerminoma (ovary) was also included in the study. Patients received a modified Einhorn regime of treatment (Einhorn & Donohue, 1977) comprising *cis*-platin $20\,mg\,m^{-2}$ i.v. for 5 days, vinblastine $0.15\,mg\,kg^{-1}$ i.v. days 1 and 2, and bleomycin 30 mg i.m. on day 2, 9, 16, the whole cycle being repeated every three weeks. Four litres

Correspondence: J.C. Willox
*Present address: Department of Pathology, Western Infirmary, Glasgow, G11 6NT, UK.
Received 9 January 1986; and in revised form 1 April 1986.

© The Macmillan Press Ltd., 1986

of 0.9% saline were administered over each 24 h and mannitol was used as required to maintain a diuresis of greater than 100 ml $h^{-1}$. Four cycles of treatment were given and patients were then reassessed, according to the criteria described in the EORTC urology group trial 30824 in which all patients were entered. Magnesium status was assessed prior to treatment by giving a loading dose of magnesium sulphate (0.25 mmol $kg^{-1}$ body weight) in 250 ml 0.9% saline over 1 h and collecting urine for 24 h. Excretion of more than 80% of the loading dose of magnesium was assumed to indicate normal body stores, and patients were stratified depending on their magnesium excretion and then randomised to receive magnesium supplements or no supplements unless the serum magnesium fell to less than 0.45 mmol $l^{-1}$.

The level of supplementation was based on the recommended daily intake of 8 mmol magnesium (DHSS, 1979) which was administered as magnesium sulphate (8 mmol in 500 ml 0.9% saline i.v. over 4 h following *cis*-platin administration, daily for 5 days). Oral supplements of magnesium citrate 10 mmol t.i.d. were commenced once the i.v. infusion was discontinued, and these supplements were continued until the next admission for intravenous treatment. A magnesium citrate syrup was formulated at Strathclyde University and prepared by Pharmacy Department, Gartnavel General Hospital. Thirty mmol of magnesium administered orally daily was estimated to provide patients with the recommended daily intake allowing for 30% absorption (Wacker & Parisi, 1968). No attempt was made to provide a placebo arm of supplements; however all clinical and biochemical assessments of patients were made by staff who were not aware at the time of whether or not the patients were receiving supplements.

Creatinine clearance was measured before treatment and was repeated prior to each course of chemotherapy. Serum sodium, potassium, creatinine, and magnesium were measured daily during in-patient treatment; liver function tests, serum calcium, plasma zinc and the tumour markers human chorionic gonadotrophin and alphafetoprotein were measured weekly. Sequential urine collections (12 h) were made during the 5 day in-patient treatment and were analysed for NAG activity and magnesium concentration. Twenty-four hour dietary recall histories with computer analysis were used to give an indication of how energy and nutrient intake changed during and between cycles (Trotter *et al.*, 1981).

### Results

Seventeen patients were studied in detail; the M:F ratio was 16:1. Eight patients were randomised to receive magnesium supplements, nine patients did not receive supplements. Ages in the supplemented group ranged from 18–39 years (mean 29 years), in the non supplemented group ages ranged from 22–47 years (mean 33 years). No patients were magnesium deficient prior to treatment. One patient in the supplemented group and two in the non supplemented group had received pre-treatment radiotherapy. Patients in each group received similar total amounts of *cis*-platin (815 mg for supplemented group; 811 mg for non supplemented group). The extent of metastatic disease was similar in each group.

Table I summarises the main findings. There was no significant difference in the numbers of complete and partial responders to treatment in each group. No patient developed clinical signs of hypomagnesaemia since intravenous or oral supplements

**Table I** Effects of magnesium supplementation after 4 cycles *cis*-platin treatment

| *Outcome* | *Supplemented patients (8)* | | *Non-Supplemented patients (9)* | |
|---|---|---|---|---|
| A. Low volume metastases | | | | |
| complete response | 5/5 | 100% | 5/5 | 100% |
| partial response | — | | — | |
| died | — | | — | |
| B. High volume metastases | | | | |
| complete response | 1/3 | 33% | 1/4 | 25% |
| partial response | 2/3 | 67% | 2/4 | 50% |
| died | — | | 1/4 | 25% |
| Patients with treatment delays | 3 | 38% | 6 | 67% |
| Patients with neutropenic episodes | 6 | 75% | 8 | 90% |
| Patients with septicaemic episodes | 2 | 25% | 2 | 22% |
| Patients with anaemia requiring transfusion | 2 | 25% | 2 | 22% |

were given when the serum magnesium fell to 0.45 mmol l$^{-1}$. Tumour markers decreased at similar rates in each group indicating no difference in response to treatment. There was a trend towards fewer treatment delays in the magnesium supplemented group, although this was not statistically significant. Neutropenic episodes which are often the cause of delaying treatment showed a similar, but not statistically significant trend. Septicaemic episodes and the frequency of anaemia requiring transfusion of blood occurred with similar frequency in each group. Myelosuppression with neutropenia and anaemia was more common in those patients from both groups who had received pre-treatment radiotherapy.

The magnesium data are summarised in Table II. The serum magnesium data are shown in more detail in Figure 1. Serum magnesium concentration showed a significant difference ($P<0.01$) between the lowest recorded levels in supplemented patients and non-supplemented patients. Although the lowest recorded serum potassium levels in the two groups were not significantly different, all patients in the non supplemented group required potassium supplementation whereas only two patients in the magnesium supplemented group required potassium supplements at any time during the four cycles of treatment. Serum calcium remained within the reference range for both groups and liver function tests were satisfactory throughout. There was no significant change in plasma zinc. Mean urine NAG activity during each course of *cis*-platin treatment is summarised in Table III. Renal tubular damage as assessed by urine NAG increased as treatment continued in the non-supplemented group. Using a non-parametric *t*-test urine NAG activity in the non supplemented group was significantly higher than the supplemented group ($P<0.01$) by the third course of treatment. This has previously been reported by our group (McAllister *et al.*, 1985). Creatinine clearance and serum creatinine levels showed no significant changes between groups and over the four courses of treatment. Urine magnesium excretion was consistently higher in the supplemented group (mean difference 6.6 mmol 24 h$^{-1}$), but less than the magnesium supplements given (8 mmol 24 h$^{-1}$).

**Figure 1** Lowest serum magnesium concentration during each course of treatment. ○, Non-supplemented group; ⊘, Non-supplemented group but received magnesium supplements; ●, Supplemented group and ●, Supplemented group but missed magnesium supplements.

Dietary energy intake in both groups showed a progressive fall from day 3 of *cis*-platin treatment, slowly increasing for 10–12 days following treatment to normal (Figure 2). There was a similar pattern for all nutrients. Treatment restarted at day 16 of the post-treatment cycle, leaving few days of improved intake to compensate for the 14 days of reduced intake. Dietary intake did not differ significantly between groups and showed even

**Table II** Serum and urine magnesium during magnesium supplementation

| Course number | *Lowest serum magnesium (mean ± s.d.) mmol l$^{-1}$* Without supplements | With supplements | *Urine magnesium excretion (mean ± s.d.) mmol 24 h$^{-1}$* Without supplements | With supplements |
|---|---|---|---|---|
| 1 | 0.67 ± 0.08 | 0.70 ± 0.06 | 3.3 ± 0.5 | 9.8 ± 1.4 |
| 2 | 0.62 ± 0.13 | 0.66 ± 0.09 | 3.1 ± 0.5 | 10.6 ± 1.0 |
| 3 | 0.50 ± 0.07[a] | 0.62 ± 0.09[a] | 3.2 ± 0.7 | 9.5 ± 1.0 |
| 4 | 0.51 ± 0.13 | 0.60 ± 0.12 | 3.3 ± 0.9 | 9.4 ± 0.6 |

[a] = $P<0.01$.

**Table III** Urine NAG excretion during *cis*-platin treatment

| | Urine NAG activity (mean ± s.d.) (x upper limit reference range) | | |
|---|---|---|---|
| *Course number* | *Without supplements* | *With supplements* | *Significance relative to course 1* |
| 1 | 2.2 ± 1.7 | 1.7 ± 1.9 | |
| 2 | 2.4 ± 0.9 | 1.8 ± 1.1 | NS |
| 3 | 3.2 ± 1.9 | 1.3 ± 0.4 | $P<0.01$ |
| 4 | 3.0 ± 1.5 | 1.9 ± 1.1 | [a] |

[a]See text. NS = not significant.

**Figure 2** Comparison of energy intakes between cycles 1 and 4 of *cis*-platin treatment (●——●, cycle 1; ×——×, cycle 4).

further fall (although not statistically significant) in course 4 compared to course 1. Intake correlated with nausea, vomiting and anorexia all of which were maximal during treatment and for two weeks following treatment. Again these did not differ between groups. Weight losses did not differ significantly between the groups with the supplemented group showing a range of 8–12.5 kg (mean, 10 kg) and the non-supplemented group showing a range of 2–25 kg (mean, 11 kg). One patient in the supplemented group gained weight (3 kg). No side effects of magnesium supplements were reported by patients, although the oral magnesium citrate syrup was pronounced 'bitter' 'metallic' and 'unpleasant to take'. Several patients stopped taking oral supplements for these reasons. There was no gastrointestinal upset associated with the supplements.

### Discussion

Supplementation of magnesium in cancer patients receiving *cis*-platin appears to be beneficial with reduced renal tubular damage and fewer treatment delays. There is no evidence of increased tumour growth, poorer outcome, or any other harmful effects to patients receiving magnesium supplements.

Previous studies on our group of cancer patients report a 43% incidence of anorexia (Willox *et al.*, 1984) plus a 73% incidence of deficiencies of all dietary components with a 78% incidence of specific dietary magnesium deficiency in out patients (Trotter *et al.*, 1981) and an 80% incidence in in-patients (Willox, 1984). A 37% incidence of low serum magnesium levels has been shown in this group (Willox, 1984). These background data, together with the marked anorexia and weight loss found in patients receiving *cis*-platin, increase the probability of these patients developing serum and body magnesium deficiencies during treatment. A retrospective review of our patients treated with *cis*-platin revealed that all patients developed hypomagnesaemia (McAllister *et al.*, 1981).

Macaulay *et al.* (1982) proposed magnesium supplementation during in-patient intravenous chemotherapy only; however this present study showed progressive decrease in serum magnesium levels as treatment continued in all patients, including those receiving intravenous and oral supplements. The optimum level of supplementation, and finding an acceptable and palatable oral supplement were beyond the scope of this study. However, the significant difference in serum magnesium levels between those on and those off supplements, and the lower serum magnesium levels found in those patients supplied with oral supplements which they were not taking points to continuous supplementation as the method of choice. It is probable that the difference between the two groups would have been considerably greater if 6 of the 8 non-supplemented patients had not received supplements when the concentration of magnesium in serum decreased to 0.45 mmol $l^{-1}$ by course 3 or 4. Short periods of intravenous supplements are unlikely to be able to compensate for the longer periods of poor dietary intake; instead they are likely to lead to an increase in urinary magnesium loss while keeping the serum magnesium levels temporarily within the normal reference range. Oral supplementation may well also be required. It should be noted that the non-supplemented group continued to excrete similar amounts of magnesium despite low concentrations of magnesium in serum. The patients appear to have an obligatory urine magnesium loss, perhaps due to the high fluid load, perhaps due to drug damage. Schilsky *et al.* (1982) have shown prolonged hypomagnesaemia following *cis*-platin treatment and long term follow up of magnesium supplemented patients in Glasgow will be interesting.

The number of patients included in this study was unfortunately small due to the number of patients referred for chemotherapy and the time available for the study to be performed. As a result it is impossible to assess the effects of supplementation in patients with low and high volume metastases separately. Although magnesium supplementation appears to be beneficial for all patients, it is likely that the greatest benefit will be to patients with large volume metastatic disease who may require prolonged treatment with *cis*-platin. Minimising treatment delays and renal damage in these patients is essential.

Magnesium supplementation should however become a routine part of *cis*-platin therapy. Further studies to examine the best methods of supplementation are indicated.

We wish to thank the medical and nursing staff and senior dietitian Mrs Jean Corr, of the Department of Oncology, Gartnavel General Hospital, Glasgow for their help in the smooth running of this study.

J.C.W. acknowledges the Cancer Research Campaign and Professor K.C. Calman who awarded her a research fellowship of which this study was a part.

### References

DEPARTMENT OF HEALTH AND SOCIAL SECURITY (1979). Recommended daily amounts of food, energy and nutrients for groups of people in the United Kingdom. HMSO: London.

EINHORN, L.H. & DONOHUE, J. (1977). *Cis*-diamminedichloroplatinum, vinblastine and bleomycin combination chemotherapy in disseminated testicular cancer. *Ann. Intern. Med.*, **87**, 293.

MACAULAY, V.M., BEGENT, R.H.J., PHILLIPS, M.E. & NEWLANDS, E.S. (1982). Prophylaxis against hypomagnesaemia induced by *cis*-platinum combination chemotherapy. *Cancer Chemother. Pharmacol.*, **9**, 179.

McALLISTER, E.J., WILLOX, J.C., CATHCART, S. & CALMAN, K.C. (1985). Reduction of *cis*-platinum nephrotoxicity following supplementation with magnesium. In *Renal Heterogeneity and Target Cell Toxicity*, Bach & Lock (eds) p. 411. John Wiley & Sons Ltd: Chichester.

McALLISTER, E.J., WILLOX, J.C., MacALISTER, A. & CALMAN, K.C. (1981). Metabolic complications of *cis*-platin chemotherapy. *J. Clin. Chem. Clin. Biochem.*, **19**, 767.

OHNUMA, T. & HOLLAND, J.F. (1977). Nutritional consequences of cancer chemotherapy and immunotherapy. *Cancer Res.*, **37**, 2395.

PARSONS, F.M., EDWARDS, G.F., ANDERSON, G.K. & 4 others (1974). Regression of malignant tumours in magnesium and potassium depletion induced by diet and haemodialysis. *Lancet*, **i**, 243.

SCHILSKY, R.L. & ANDERSON, T. (1979). Hypomagnesaemia and renal magnesium wasting in patients receiving *cis*-platin. *Ann. Intern. Med.*, **90**, 929.

SCHILSKY, R.L., BARLOCK, A. & OZOLS R.F. (1982). Persistent hypomagnesaemia following *cis*-platin chemotherapy for testicular cancer. *Cancer Treat. Rep.*, **66**, 1767.

STUART-HARRIS, R., PONDER, B.A.J. & WRIGLEY, P.F.M. (1980). Tetany associated with *cis*-platin. *Lancet*, **ii**, 1303.

TROTTER, J.M., DUFFY, J., CALMAN, K.C. & WILLOX, J.C. (1981). Dietetic evaluation of cancer patients. *Br. J. Cancer*, **44**, 292.

WACKER, W.E.C. & PARISI, A.F. (1968). Magnesium metabolism. *N. Engl. J. Med.*, **278**, 658, 712, 772.

WILLOX, J.C., McALLISTER, E.J., CLARK, S., TROTTER, J.M. & CALMAN, K.C. (1981). Hypomagnesaemia following chemotherapy. *Cancer Topics*, **3**, 64.

WILLOX, J.C., CORR, J., SHAW, J., RICHARDSON, M., CALMAN, K.C. & DRENNAN, M. (1984). Prednisolone as an appetite stimulant in cancer patients. *Br. Med. J.*, **288**, 27.

WILLOX, J.C. (1984). Metabolic and nutritional implications of cancer and chemotherapy. *M.D. thesis*, Glasgow University.